# Here Comes Hypercar!

**DOI:** 10.1289/ehp.113-a250

**Published:** 2005-04

**Authors:** David C. Holzman

Despite the fact that the price of gasoline seems stuck around $2.00 per gallon, gas-guzzling SUVs and pickups remain as popular as ever among Americans. The United States produces 25% of the world’s greenhouse gas emissions; cars and light trucks account for around 20% of the nation’s energy-related greenhouse gas emissions, according to the U.S. Energy Information Administration. It would appear that a gas-saving, nonpolluting car for the U.S. masses will need to be something that even car enthusiast magazines could applaud. It will need to equal or beat conventional cars in handling, performance, size, safety, and amenities, and do so at a competitive cost. It’s a tall order, but Amory Lovins, chief executive officer of the nonprofit Rocky Mountain Institute (RMI) in Snowmass, Colorado, thinks he may have just the car to meet it: the Hypercar^®^.

The latest version of Lovins’s Hypercar concept is a detailed virtual design illustrating an SUV crossover vehicle that would fully compete with today’s midsize entry-level luxury SUVs. Powered by a hybridized hydrogen fuel cell, the concept, dubbed Revolution, would achieve Environmental Protection Agency mileage ratings equivalent to 108 miles per gallon (mpg) of gasoline; as a Prius-like gasoline hybrid, 62 mpg, and with a good nonhybrid gasoline engine, 45 mpg, according to extensive RMI simulations.

The gasoline hybrid version could sell profitably for $40,000–45,000 (in year 2000 dollars), at standard markups based on what Lovins calls extensive supplier price quotations for 82% of the components, plus bottom-up cost modeling by RMI and independent consultants for technologies not yet in production. Further development could pare the price to about $35,000.

But Lovins’s concept goes beyond transportation. A national fleet of fuel cell–powered Hypercar-class vehicles could contribute to the national electricity grid when they are parked—which averages about 96% of the time, according to the Population Reference Bureau. And according to RMI, if Hypercars captured half of the world’s market by 2020, global carbon dioxide emissions from cars and trucks would fall 25%, instead of rising by 12%. (This estimate assumes that the efficiency of conventional cars improves by 25%, and vehicle miles traveled increases by 50%.)

## Light Years Ahead?

Lovins’s big idea that makes the Hypercar seem attainable is what he calls “the snowballing of weight savings.” Essentially, if you lose enough weight by trading a car’s steel body for composite fiber, then power requirements will drop. The engine, drive train, and suspension can all be less massive, reducing weight still further. At this point, systems such as power steering and power brakes may become superfluous; the need for such systems is largely a function of weight. At 1,887 pounds, the five-seat Revolution would weigh less than half as much as a conventional counterpart and about the same as the two-seat aluminum Honda Insight, currently one of the most fuel-efficient vehicles in the United States.

All this lightweighting has yet another benefit. Fuel cells are so expensive today—*Consumer Reports* put the price at around $19,000 in 2004—that they are commonly viewed as a couple of decades from practical automotive application. But the combination of light weight, streamlining, and low-friction tires would enable the Revolution to cruise at 55 miles per hour (mph) on the same power to the wheels that a normal SUV uses on a hot day just to run its air conditioner, according to Lovins. That means the car could use a fuel cell stack one-third the size needed for a comparable conventional light-duty vehicle. Plus, hydrogen storage tanks that are already available could be used in the cars, providing a range of 330 miles before refueling.

The Revolution power train integrates a 35-kilowatt (47 horsepower) ambient-pressure fuel cell, 35-kilowatt nickel metal hydride buffer batteries, and four electric motors connected to the wheels with single-stage reduction gears. The batteries store energy captured through regenerative braking, meaning that when you apply the brakes, a generator does the braking, recharging the batteries. This provides extra oomph for fast acceleration, climbing hills while loaded, and other bursts of energy.

Twenty-five percent of the Revolution’s weight reduction over comparable conventional cars is achieved by building the body from carbon fiber–reinforced composite. The enormous strength of carbon fiber composites can make cars extremely safe. Drivers have walked away from 200-mph crashes in ultralight carbon fiber Formula One race cars. The Revolution is designed to protect passengers from serious injury in a 30-mph head-on crash with a vehicle twice its weight.

Carbon fiber structures can absorb five or more times the energy per pound as steel, and can do so more smoothly. Metal absorbs crash energy by bending and folding; a 1-foot tube of aluminum might fold 8 times until it’s fully compressed. Carbon fiber structures, on the other hand, sustain microscopic cracks; a foot of composite might sustain 10,000 microcracks, each essentially representing a unit of energy absorption. The front end of the Revolution is a welded-aluminum tubular structure that incorporates some composite crush structure as well, and the vehicle is designed so that damaged material can be removed and replaced.

## Hypercar Hurdles

The Hypercar’s light weight does present a couple of challenges. One has to do with the ratio of the fully loaded car to the empty car, which would bearound 1.5 to 1—not all that different from pickup trucks used for commercial hauling. With a traditional suspension, Hypercars could, like pickups, tend to bounce around on the road when empty.

To deal with this problem, the Hypercar would have a “semi-active” suspension. It would be sprung on air, and a compressor would increase stiffness as needed. The shock absorbers would be linear motors that could be adjusted for a firmer or softer suspension. The shock absorbers would also be able to recover electrical energy from going over bumps.

The carbon fiber structures also face technical and economic hurdles. One problem is the long time it takes to fabricate parts out of carbon fiber. One solution is to stamp thermoplastic composites (a type of carbon fiber material), which can take less than 1–2 minutes. A composite sheet would be heated up and shuttled into a press, and stamped into shape similar to the way sheet metal is stamped out.

Nonetheless, 1–2 minutes is still a long cycle time, says David Cole, chairman of The Center for Automotive Research in Ann Arbor, Michigan. “The economics are tightly entwined with the speed of the process,” he says. “You would have to have a lot more machinery and dies because of the low volume of any one part of the process. That becomes a real concern.”

Yet, speed isn’t everything, says Lovins. Composites can be molded into a single complex part, and the composite manufacturing methods are more conducive to forming complex parts. In contrast, metal parts typically are made from several stamped parts of relatively simple shapes that are then welded together to form the complex shape desired. The body of a Revolution would include 65% fewer major parts and 77% fewer total parts than a comparable conventional steel body, and molding each composite part would need one die set compared to the average of four needed to stamp steel, says Lovins. Plus, the composite parts would have color molded into them, eliminating the need for painting the vehicle.

At volume, Lovins believes such characteristics can make automaking two-fifths less capital-intensive than today’s leanest plant. According to the 2004 RMI report *Winning the Oil Endgame*, once you take into account the simpler assembly, the eliminated paint shop, and the smaller propulsion system, the extra per-car cost of the Hypercar drops to approximately zero.

Cole sees several economic and cultural hurdles to the Hypercar concept, but none that can’t be overcome. Besides the economics of panel fabrication, these hurdles include a current lack of body repair shops that can handle carbon fiber composites, and the inertia of an industry that has enormous capital invested in conventional methods. The car industry has to see something to believe it, says Cole. “[Manufacturers] really want to see it demonstrated,” he says. “[But] if Amory can do what he thinks he can do, it will turn the world upside-down.”

## Hydrogen Transition

If the value of hydrogen fuel cell–powered cars extended beyond transportation, it could possibly hasten their adoption. According to Brett Williams, a researcher and Ph.D. candidate at the University of California, Davis, Institute of Transportation Studies, these cars could provide power companies with “spinning reserves”—a term that comes from the image of power-generating turbines spinning disconnected from the grid, ready to be brought online when needed. The cars could also be plugged into the electric grid while their drivers work—initially, motorists might even be paid for their cars’ services as generators in special power-generating parking lots, says Williams.

One oft-cited hurdle to alternative vehicle use is the chicken-and-egg problem of cars and filling stations—you can’t have the hydrogen cars without the hydrogen filling stations, and you can’t have the filling stations without the cars. In response to this obstacle, Lovins points to a self-financing solution he and Williams presented in 1999 at the 10th annual National Hydrogen Association meeting and outlined in their paper titled “A Strategy for the Hydrogen Transition.” In this strategy, fuel cells would be adopted to provide power not only to vehicles but also to homes, office buildings, and other settings. Retail refueling stations would produce hydrogen from natural gas. The result, ultimately, would be an interactive, self-regenerative hydrogen infrastructure. Lovins and Williams write that the strategy relies on existing technologies, can begin immediately, and proceeds in a logical and viable sequence.

The capital intensity of such a hydrogen refueling infrastructure is probably less than the capital intensity of sustaining the existing gasoline fueling infrastructure, according to Lovins. “Hydrogen can be used so much more efficiently than hydrocarbons,” he says, “that reforming natural gas into hydrogen scarcely increases its demand, [when you account for] the resulting savings in refineries, power plants, furnaces, and boilers.”

## Figures and Tables

**Figure:**
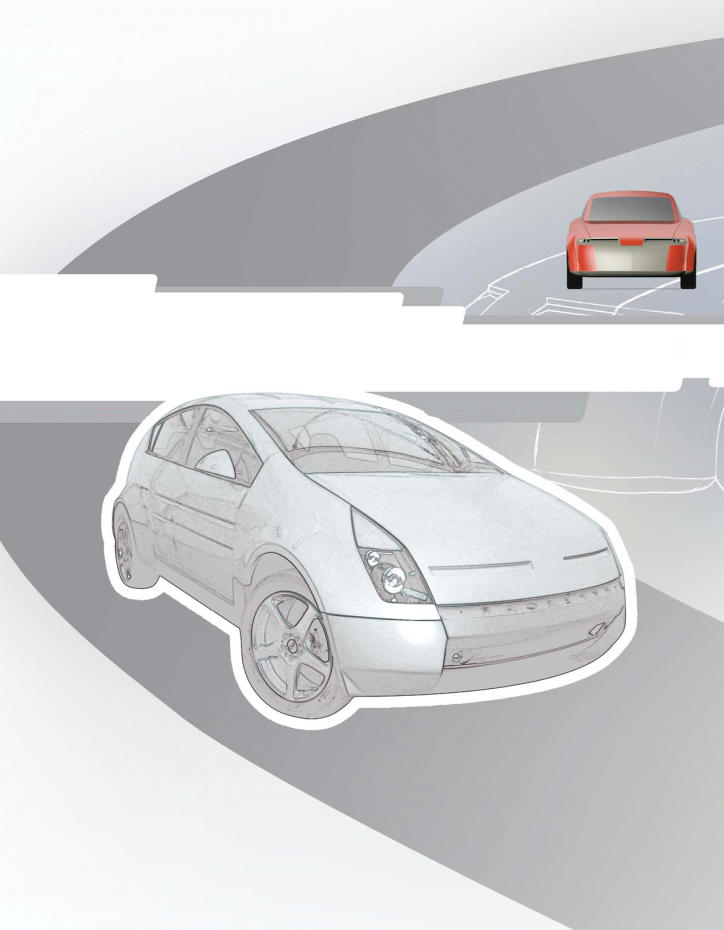


**Figure:**
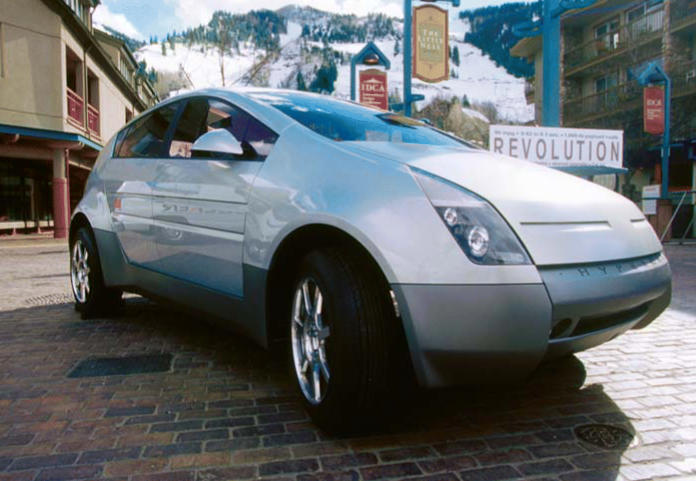
Stopping traffic The 2000 Hypercar Revolution models the virtual design of a midsize SUV. The carbon fiber body makes it lighter, safer, and—at 114 mpg with a fuel cell—far more fuel-efficient than traditional cars.

**Figure:**
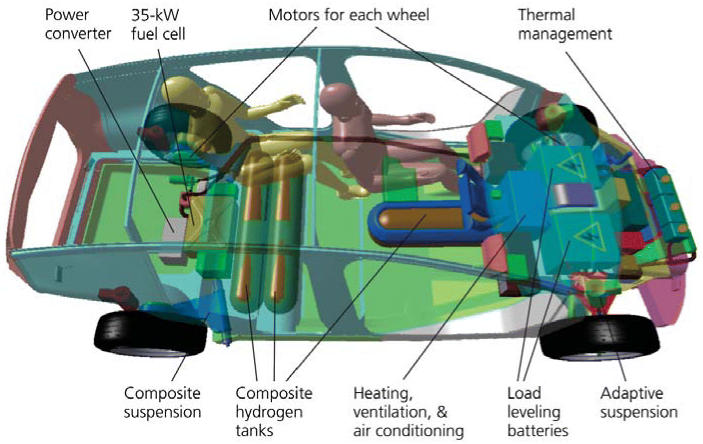
Dream car dissected Ultralight vehicles suggest a solution to the problem of storing hydrogen for fuel cell–powered engines. A diagram of the Revolution concept SUV shows that efficiency-tripling platform physics can shrink the hydrogen tanks by threefold. The three tanks shown in this design provide a 330-mile average driving range on 3.4 kilograms or 138 liters of hydrogen at 5,000 pounds per square inch, yet allow an interior that can hold five adults and up to 69 cubic feet of cargo space with the rear seats folded flat. Such tanks have been demonstrated to be highly crashworthy, in part because they’re supported by interior pressure. The transverse tanks in this design have room to move axially in a side-impact collision. The fuel cell also becomes three times smaller and more affordable.

## References

[b1-ehp0113-a00250] Congressional Budget Office 2002. Reducing Gasoline Consumption: Three Policy Options. Washington, D.C.: Congressional Budget Office. Available: http://www.cbo.gov/showdoc.cfm?index=3991&sequence=0 [accessed 8 March 2005].

[b2-ehp0113-a00250] Lovins AB Datta EK Bustnes OE Koomey JG Glasgow NJ 2004. Winning the Oil Endgame: Innovation for Profits, Jobs, and Security. Snowmass, CO: Rocky Mountain Institute. Available: http://www.oilendgame.org/pdfs/WtOEg_72dpi.pdf [accessed 8 March 2005].

[b3-ehp0113-a00250] Lovins AB Williams BD 1999. A Strategy for the Hydrogen Transition. Snowmass, CO: Rocky Mountain Institute. http://www.rmi.org/images/other/Trans/T99-07_StrategyH2Trans.pdf [accessed 8 March 2005].

